# Receptor activator of nuclear factor kappa-B ligand (RANKL) but not sclerostin or gene polymorphisms is related to joint destruction in early rheumatoid arthritis

**DOI:** 10.1007/s10067-017-3570-4

**Published:** 2017-02-11

**Authors:** Antonia Boman, Heidi Kokkonen, Lisbeth Ärlestig, Ewa Berglin, Solbritt Rantapää-Dahlqvist

**Affiliations:** Department of Public Health and Clinical Medicine/Rheumatology, Umeå University, SE-901 85 Umeå, Sweden

**Keywords:** Early rheumatoid arthritis, Radiological progression, Receptor activator of nuclear factor kappa-B ligand, Sclerostin, Single nucleotide polymorphism

## Abstract

The aim of this study was to analyze relationships between receptor activator of nuclear factor kappa-B (RANKL), sclerostin and their gene polymorphisms with radiological progression in patients with early rheumatoid arthritis (RA). Patients with early RA (*n* = 407, symptomatic <1 year) (ARA criteria) examined radiologically at inclusion and after 24 months were consecutively included. Disease activity score and C-reactive protein were regularly recorded. Sclerostin, RANKL, and anti-CCP2 antibodies were analyzed in plasma at baseline using ELISAs. Data on gene polymorphism for sclerostin and RANKL were extracted from Immunochip analysis. Sex- and age-matched controls (*n* = 71) were identified from the Medical Biobank of Northern Sweden. The concentration of RANKL was significantly higher in patients compared with controls, median (IQR) 0.56 (0.9) nmol/L and 0.20 (0.25) nmol/L (*p* < 0.001), and in anti-CCP2-positive patients compared with sero-negative individuals. Sclerostin was significantly increased in female patients 0.59 (0.47–0.65) ng/mL compared with female controls 0.49 (0.4–0.65) ng/mL (*p* < 0.02). RANKL concentration was related to the Larsen score at baseline (*p* < 0.01), after 24 months (*p* < 0.001), and to radiological progression at 24 months (*p* < 0.001). Positivity of RANKL and anti-CCP2 yielded significant risk for progression with negativity for both as reference. No single nucleotide polymorphism encoding TNFSF11 or SOST was associated with increased concentrations of the factors. The concentration of RANKL was related to the Larsen score at baseline, at 24 months, and radiological progression at 24 months particularly in anti-CCP2-positive patients, while the concentration of sclerostin was unrelated to radiological findings*.*

**Electronic supplementary material:**

The online version of this article (doi:10.1007/s10067-017-3570-4) contains supplementary material, which is available to authorized users.

## Introduction

Rheumatoid arthritis (RA) is a chronic autoimmune disease characterized by joint inflammation that eventually leads to the destruction of cartilage and bone (1). The destructive process is related to the presence of autoantibodies, genetic polymorphism involving proteins in the Wingless (Wnt)-β-catenin pathway, and markers of inflammation, cartilage and bone metabolism (2, 3).

Receptor activator of NF kappa B Ligand (RANKL) is a member of the tumor necrosis factor (TNF) family of cytokines and is coded for by the tumor necrosis factor ligand superfamily member 11 (*TNFSF11*) gene. The protein plays a central role in osteoclast differentiation and activation and the RANKL/osteoprotegerin pathway (RANKL/OPG) is strongly upregulated by pro-inflammatory cytokines (4). This is an important pathway for inflammatory bone loss in patients with RA (5). RANKL is not only expressed in osteocytes and osteoblasts but also in synovial cells, activated T cells, B cells, and natural killer cells (4). Synovial cells expressing RANKL are responsible for the formation of osteoclasts and bone loss in an experimental model of RA directly linking the immune system to bone (6). Furthermore, bone erosions can be retarded in RA patients by clinical blockage of RANKL (7).

Sclerostin is an osteocyte-specific protein that is a product of the SOST gene and is a potent suppressor of bone formation (8). Sclerostin inhibits the Wnt-signaling pathway, thereby blocking osteoblast formation and inhibiting the production of bone (9). Sclerostin binds to the low density receptor, lipoprotein receptor-related protein 5 (LRP5), and promotes Wnt-blocking by releasing intra-cellular β-catenin to the cytoplasm that ultimately leads to osteoblast differentiation (3). In patients with RA, sclerostin may be responsible for the low level of bone repair, and inhibition of the protein could effectively increase repair of bone erosions in experimental arthritis (10).

Joint destruction as a measurement of the severity of RA is evaluated by the extent of radiologically detected progression of joint damage. It is important to identify those patients with rapidly progressive joint destruction in order to initiate a more aggressive treatment regimen (11). Useful biomarkers for the severity of disease progression are currently sparse in patients with RA; as a consequence, patient treatment is rarely individualized. Currently used models in clinical practice to predict the destructive disease course involve degradation products of cartilage or bone, rheumatoid factor (RF), anti-citrullinated protein antibodies (ACPA), and/or measurements of inflammatory activity [e.g., DAS28, C-reactive protein, erythrocyte sedimentation rate (ESR)] (11–13). However, there is a need for more biomarkers to increase the reliability of analyses to predict the disease progression.

In this study of radiological findings at baseline and after 24 months in patients with early RA, we have evaluated baseline values of RANKL and sclerostin as potential biomarkers for assessing a more aggressive disease course. We also aimed to investigate the relationships between gene polymorphisms of the two proteins, sclerostin and RANKL, extracted from the Immunochip data, and plasma concentrations for radiological progression.

## Materials and methods

### Patients and controls

A total of 407 patients (69% female) with early RA (i.e., the duration of symptoms <1 year) (14) who underwent radiological examination at inclusion and after 24 months were consecutively included in the study. These patients belong to a prospective inception cohort study, but for this study, only individuals with radiological examinations at baseline and consistently performed after 24 months were included. Disease activity score (DAS28) was calculated at baseline and after 6, 12, 18, and 24 months using the 28-joint count of tender (TJC) and swollen joints (SJC), the patient’s global assessment, and erythrocyte sedimentation rate (ESR, mm/h) (15). The concentrations of ESR, C-reactive protein (CRP, mg/L), and of RF as Waaler-Rose hemagglutination test with sensitized sheep red blood cells for RF were analyzed according to routine clinical protocols. Sensitivity analyses of baseline data between the whole inception cohort and the cohort with x-ray data as included in this study did not show any significant differences between the groups concerning DAS28, CRP, ESR, SJC, and TJC or the treatments. The patients were treated during the 24 months with the aim of achieving remission by using disease-modifying anti-rheumatic drugs (DMARDs) or corticosteroids with respect to the clinical situation identified by the patients’ physician. During the first 24 months (mean ± SEM duration, 9.8 ± 0.5 months), 52.3% were treated with corticosteroids (mean dose ± SEM, 6.9 ± 0.2 mg/day), 98.3% with DMARDs [in 88.1% methotrexate (mean ± SEM duration, 20.4 ± 0.4 months; mean dose ± SEM, 18.2 ± 1.9 mg/week orally or injectable), 39.1% sulfasalazine (dose 1000–2000 mg/day), 28.7% chloroquine (dose 200–400 mg/day), 9.1% myocrisine (10–50 mg/week), 5.2% azathioprine (50–150 mg/day), 2.2% cyclosporin (100–175 mg/day), 2.0% leflunomide (10–20 mg/day), and as combination therapy in 36.8%], and 9.1% with biologics (adalimumab, etanercept, infliximab). Response to treatment was evaluated at 6 and 24 months using EULAR response criteria (16).

Blood samples were collected from patients at baseline, i.e., when presenting with early RA. As controls, a total of 71 (81.7% female) individuals were collected from the Medical Biobank of Northern Sweden and matched, from the same geographical area as the patients, for sex and range of age at a group level. Patients and controls were classified either as being a “non-smoker” or an “ever-smoker” (past or current). Anti-cyclic citrullinated peptide antibodies (anti-CCP2 antibodies) were detected using enzyme-linked immunosorbent assay according to the manufacturer’s instructions (Euro-Diagnostica AB, Malmö, Sweden) with a cut-off value for positivity at 25 AU/mL. Genotyping of HLA-DRB1 was performed as previously described and HLA-SE was defined as HLA-DRB1*0401/0404/0405/0408/0101 as previously described (19)

Descriptive data of the patients with early RA at inclusion and controls are presented in Table 1. The participants gave their written informed consent, and the Regional Ethics Committee at the University Hospital in Umeå approved the study.

**Table 1:** Descriptive data for 407 patients with early rheumatoid arthritis and for 71 controls, at the time of inclusion into the study and during follow-up until 24 months

Variables	RA patients (*N* = 407)	Controls (*N* = 71)
Age (mean) ± SD, years	53.8 ± 14.5	54.9 ± 14.5
Female, *n* (%)	281/407 (69)	58/71 (81.7)
HLA-SE, *n* (%)	242/404 (59.9)*	30/67 (44.8)
*PTPN22* 1858T carriage (%)	137/402 (34.1)	18/52 (25.7)
RF+, *n* (%)	327/407 (80.3)	–
Anti-CCP2 abs+, *n* (%)	305/407 (74.9)***	1/71 (1.4)

*HLA-SE* HLA shared epitope = 0101/0401/0404/0405/0408, *RF* rheumatoid factor, *Anti-CCP2 abs* anti-CCP2 antibodies

* *p* < 0.05, ***p < 0.001

### Evaluation of radiographs

Radiographs of the hands, wrists, and feet at baseline and after 2 years were graded according to the Larsen score by two specially trained rheumatologists (17). Radiological progression was defined as the increase of the Larsen score between baseline and 24 months, with the smallest detectable change of less than 4 calculated according to Bruynesteyn et al. (18).

### Immunoassays for sclerostin and RANKL

Human Sclerostin HS EIA Kit (TECOmedical Group, Sissach, Switzerland) was used to measure concentrations of sclerostin in plasma from patients and controls according to the manufacturer’s instructions. The range of detection was 0.165 to 2.578 ng/mL. The cut-off for sclerostin positivity was set as the 95th percentile of the controls, i.e., 1.14 ng/mL. Human RANKL ELISA (BioVendor, Karasek, Czech Republic) was used to determine the total RANKL concentrations (free and bound) in plasma performed according to the manufacturer’s instructions. The range of detection was 0.05 to 3.20 nmol/L. The cut-off for positivity of RANKL was set as the 95th percentile, i.e., 0.92 nmol/L.

### Gene polymorphism

Data on gene polymorphisms were extracted from Immunochip analysis (SNP&SEQ Technology Platform, Uppsala, Sweden) covering three single nucleotide polymorphisms (SNPs) for SOST gene and 539 for the *TNFSF11* gene (20). Information about the protein tyrosine phosphatase, non-receptor type 22 (*PTPN22*) C1858T was also extracted from the Immunochip analysis.

### Statistical analysis

For comparative analyses between continuous data, non-parametrical tests were used since the variables were not normally distributed. The Mann-Whitney *U* test was used for two groups and the Kruskal-Wallis test for more than two groups. Univariate analyses of variance were used to investigate the relative strengths of the relationships between the variables (presented in Tables 3 and 4) and radiographic outcome. Thereafter, all significant variables were included in multivariate analyses. Radiological progression was analyzed and dichotomized in relation to the SNPs. Area under the curve (AUC) was calculated for DAS28, CRP, and ESR during 6, 12, and 24 months. The concentration of RANKL was transformed to log10 as it was not normally distributed. Additive interactions were calculated as the attributable proportion (AP), the relative excess risk due to interaction (RERI) and the synergy index (SI) with confidence intervals (CI). Multiplicative interaction (MI) was assessed by adding an interaction variable  to logistic regression models. Calculations were performed using IBM SPSS Statistics (version 21.0) for Windows and statistical significance was considered as *p* ≤0.05. Genetic analyses of SNPs in relation to concentration were performed using PLINK (1.07) (21) and Haploview (4.2) for the permutation test (http://www.broad.mit.edu/mpg/haploview).

## Results

**Fig. 1:**
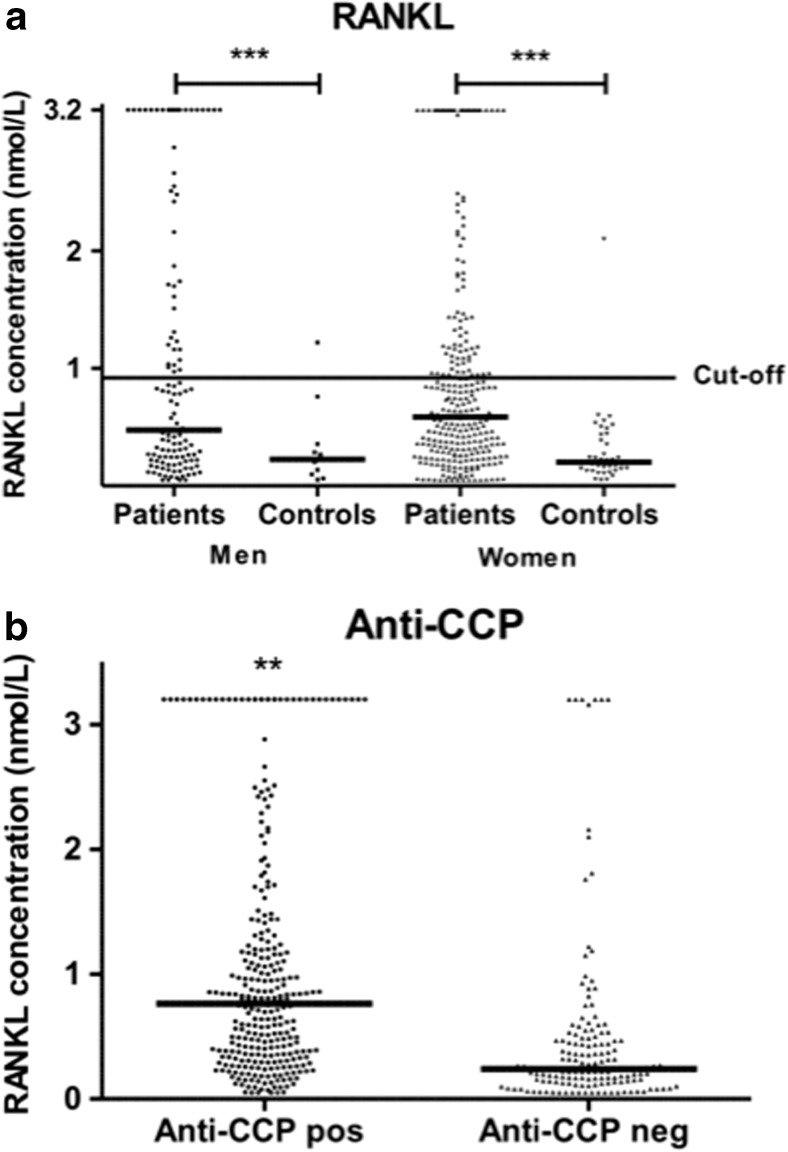
**a** Concentration of RANKL in patients and control subjects. **b** Concentration of RANKL in patients stratified for anti-CCP2 antibodies

The concentration of RANKL analyzed in samples collected at baseline was significantly increased in patients compared with controls, median (quartile 1–quartile 3) 0.56 (0.26–1.16) nmol/L and 0.20 (0.13–0.38) nmol/L, respectively (*p* < 0.001) (Fig. 1a). Anti-CCP-positive patients had significantly higher concentration of RANKL compared with anti-CCP-negative patients, median (Q1–Q3) 0.764 (0.347–1.325) nmol/L and 0.242 (0.138–0.474) nmol/L as was the concentration in RF-positive patients (Fig. 1b). A significantly increased concentration in anti-CCP-positive patients remained when analyzed in individuals sero-negative for RF (*p* < 0.02). The concentration of RANKL was related to ESR at baseline and at 12 months adjusted for age and sex (*p* < 0.01 for both) and correlated with AUC-ESR6, 12 and 24 months, respectively (correlation coefficient 0.12–0.13, *p* < 0.05 for all three), but was unrelated to DAS28, CRP, TJC, or SJC, sex, age, and smoking habits. The frequency of RANKL above cut-off (defined as above the 95th percentile of the controls’ values) was 32.9%. The presence of positivity for RANKL was significantly associated with positivity for anti-CCP2 antibodies and RF (*p* < 0.001) and with AUC-ESR6, 12 and 24 months (*p* < 0.05 for all three).

Analyses of the combinations of RANKL and anti-CCP2 (presented in Table 2) showed an increase of OR for radiological progression when being positive for both compared with being negative for both as reference. However, interaction analysis, additive or multiplicative, did not show a significant interaction between these factors (Table 2). The combinations of being both RANKL and anti-CCP2 positive yielded also significantly higher Larsen score at 24 months (data not shown).

**Table 2:** Odds ratio (OR, 95% CI) for combinations of RANKL (positive/negative) and anti-CCP2 antibodies for radiological progression at 24 months

RANKL	Anti-CCP2	Radiological progression yes/no	OR (95% CI)
−	−	27/59	1.0 (ref.)
+	−	4/11	0.80 (0.23, 2.72)
−	+	81/97	1.83 (1.06, 3.14)
+	+	60/56	2.34 (1.31, 4.20)

RERI = 0.7219148 ( −1.548815, 2.185976)

AP = 0.3083433 (−0.08292323, 0.6392927)

SI = 2.165591 (0.2539086, 18.47037)

MI = 1.614709 (0.4322704, 6.032153), *p* value = 0.476067

**Fig. 2:**
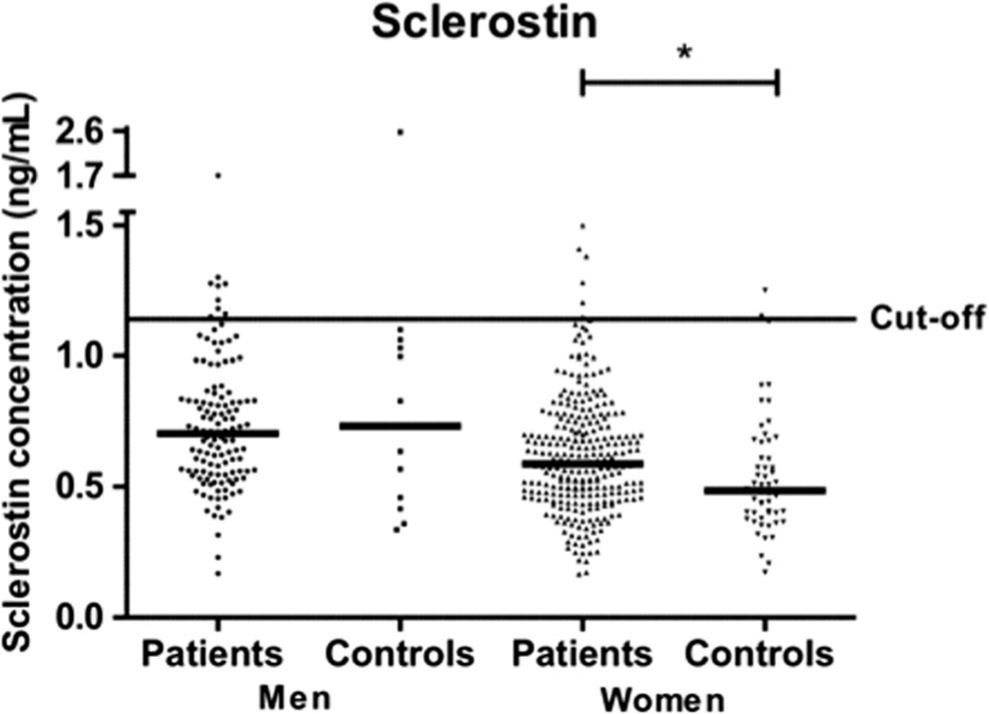
Concentration of sclerostin in patients and control subjects

The concentration of sclerostin was also significantly increased in RA patients, median (Q1–Q3) 0.63 (0.49–0.78) ng/mL versus controls 0.51 (0.4–0.7) ng/mL (*p* < 0.01) (Figs. 1c and 2). However, when stratified for sex, the levels were only significantly increased in female patients, 0.59 (0.47–0.74) ng/mL, compared with 0.49 (0.4–0.65) ng/mL in female controls (*p* < 0.02). The concentration of sclerostin was affected by age with increasing concentration in both sexes [*β* = 0.008 (95% CI 0.004, 0.012), *p* < 0.001 for males and *β* = 0.005 (95% CI 0.003, 0.007), *p* < 0.001 for females]. There were no relationships between sclerostin concentrations and anti-CCP2 antibodies or any makers of inflammation (e.g., DAS28, ESR, CRP, or joint count calculated separately or for AUC values).

Carrier of HLA-SE and the *PTPN22* T-variant were not related to the concentrations of RANKL or sclerostin. During the 24 months of the study, the DAS28 decreased significantly in the patients (*p* < 0.001) (Supplementary Table 1) while the Larsen score increased significantly (*p* < 0.001) during the corresponding time. At 6 months, 60.3% were good-moderate responders, and at 24 months, 72.5% were responders.

### Predictors of radiographic outcome: univariate analysis of variance

The concentration of RANKL and RANKL positivity, but not the log RANKL, was related to the Larsen score at inclusion as were carriage of *PTPN22* T variant and greater age at inclusion and baseline values for DAS28, ESR, and SJC (Table 3). RANKL, measured as concentration or positivity, were related to the Larsen score at 24 months, as were age, male sex, Larsen score at baseline and the presence of RF and anti-CCP antibodies, and inflammatory activity measurements at all time points as measured by CRP (*p* value <0.001–0.01), ESR (*p* value <0.001 at all time points), SJC (*p* value 0.001–0.05), DAS28 (*p* value 0.001–0.05, respectively, except for non-significant at 24 months), and response to therapy at 24 months.

**Table 3:** Univariate analyses of variance of clinical and laboratory data as potential predictors for joint destruction in patients with early RA measured at baseline and after 24 months

	Larsen score (inclusion)		Larsen score (24 months)		Radiographic progress (24 months)	
	*β* value (95% CI)	*p* value	*β* value (95% CI)	*p* value	*β* value (95% CI)	*p* value
Age at onset	0.13 (0.10–0.17)	<0.001	0.13 (0.07–0.19)	<0.001		ns
Sex		ns	2.02 (0.22–3.82)	0.028	1.74 (0.62–2.85)	0.002
Larsen score at baseline	–		1.11 (1.02–1.19)	<0.001	0.15 (0.06–0.23)	0.001
Erosions at baseline	–		6.9 (5.27–8.55)	<0.001		ns
*PTPN22* 1858T	1.40 (0.12–2.68)	0.033		ns		ns
RF positivity		ns	3.05 (0.95–5.14)	0.004	2.14 (0.84–3.44)	0.001
Anti-CCP positivity		ns	2.68 (0.76–4.6)	0.006	1.72 (0.52–2.92)	0.005
RF/anti-CCP positivity		ns	3.46 (1.37–4.96)	0.001	1.94 (0.82–3.06)	0.001
RANKL concentration (nmol/L)	0.91 (0.29–1.53)	0.004	1.8 (0.98–2.67)	<0.001	0.99 (0.45–1.52)	<0.001
RANKL positivity^a^	1.26 (0.02–2.54)	0.053	2.58 (0.82–4.33)	0.004	1.32 (0.22–2.42)	0.019
Log RANKL, concentration (nmol/L)	0.76 (−0.5–2.02)	0.234	2.38 (0.65–4.10)	0.007	0.112 (0.01–0.21)	0.032
Sclerostin concentration (ng/mL)	0.93 (−1.64–3.50)	0.477	−0.19 (−3.74–3.37)	0.92	−0.92 (−3.13 to 1.29)	0.411
Sclerostin positivity^a^	0.89 (−2.85–4.64)	0.639	−1.50 (−6.66–3.66)	0.567	−2.21 (−5.42 to 0.99)	0.176
CRP baseline		ns	0.05 (0.02–0.10)	0.004	0.03 (0.01–0.06)	0.007
ESR baseline	0.03 (0.00–0.06)	0.05	0.06 (0.02–0.10)	0.002	0.04 (0.01–0.06)	0.002
SJC baseline	0.24 (0.12–0.35)	<0.001	0.30 (0.14–0.46)	<0.001		ns
DAS28, baseline	0.59 (0.15–1.02)	0.008	0.7 (0.10–1.29)	0.022		ns
Response at 6 months		ns		ns	−1.38 (−0.31 to 2.45)	0.012
Response at 24 months		ns	−2.13 (−0.15 to 3.99)	0.024	−1.68 (−0.51 to 2.85)	0.005

*CI* confidence interval, *ESR* erythrocyte sedimentation rate, *DAS28* disease activity score, *CRP* C-reactive protein, *anti-CCP* anti-cyclic citrullinated peptide, *RF* rheumatoid factor, *TJC* tender joint count, *SJC* swollen joint count

^a^Cut-off for RANKL and sclerostin was based on above the 95th percentile of the controls

The concentration of RANKL, as both crude or log value, was also related to radiographic progression at 24 months as well as male sex, baseline values for Larsen score, RF and anti-CCP2 antibodies, and inflammatory markers from all time points except for baseline values of CRP and ESR but for response to therapy at both 6 and 24 months. Carriage of HLA-SE was not related to the radiological findings (Table 3). The levels of sclerostin were unrelated to the radiological findings.

### Multiple regression analyses of variance

Including the variables significantly related to the radiological findings in univariate analyses of variance for a multiple analyses of variance showed that only age remained significantly associated with Larsen score at baseline (Table 4). The Larsen score at 24 months was related to the RANKL concentration adjusted for Larsen score at baseline, anti-CCP2 antibodies, sex, DAS28, and response at 24 months. The radiological progression was related to the log RANKL concentration with the same adjustments (Larsen score at baseline, DAS28, anti-CCP2 antibodies, sex, and therapeutic response both at 6 and 24 months) in the multiple analyses of variance (Table 4). In all of the analyses, similar results were achieved when including RANKL concentration as crude value [β value (95% CI) for Larsen score at inclusion, 0.84 (0.23–1.46), *p* = 0.007; Larsen score at 24 months, 0.67 (0.15–1.19), *p* = 0.012; and radiological progression, 0.75 (0.21–1.28), *p* < 0.006]. C-reactive protein or ESR instead of DAS28 from the different time points at which they were collected yielded similar results as DAS28 values (data not shown). Adjustment for corticosteroid and/or DMARDs treatment (yes/no) or duration of treatment did not affect the results (data not shown).

**Table 4:** Multivariate analyses of variance including factors of potential predictors for joint destruction in patients with early RA measured at baseline and 2 years

	Larsen score at inclusion^a^		Larsen score at 24 months		Radiological progression at 24 months	
	*β* value (95% CI)	*p* value	*β* value (95% CI)	*p* value	β value (95% CI)	*p* value
Age at onset, years	0.108 (0.066–0.149)	<0.001	–		–	
Sex	–		2.10 (1.03–3.17)	<0.001	2.05 (0.96–3.14)	<0.001
Larsen, baseline	–		1.09 (1.00–1.17)	<0.001	0.13 (0.05–0.21)	0.002
Anti-CCP positivity		ns	1.35 (0.15–2.54)	0.028	1.37 (0.15–2.58)	0.027
Log RANKL concentration (nmol/mL)	0.52 (−0.79 to 1.84)	0.43	1.11 (0.03–2.19)	0.045	1.20 (0.10–2.30)	0.033
DAS28 baseline	0.38 (−0.04 to 0.80)	0.075	0.36 (−0.01 to 0.72)	0.056	0.48 (0.09–0.86)	0.016
Response at 6 months	–		–		−1.29 (−0.24 to 2.33)	0.016
Response at 24 months	–		−2.35 (−1.23 to 3.47)	<0.001	−2.1 (−0.94 to 3.24)	<0.001

^a^Adjustment: *PTPN22* T variant. *CI* confidence interval, *DAS28* disease activity score, *anti-CCP* anti-cyclic citrullinated peptide

The associations of SNPs of *TNFSF11* and *SOST*, extracted from the Immunochip, respectively were determined by linear regression analysis using PLINK. Of the *TNFSF11* gene, 50 SNPs were associated with the concentration of RANKL (*p* < 0.003–0.05, uncorrected). All SNPs from the Immunochip were located upstream of the coding region of the gene. After permutation test as a correction for multiple testing, no association between any of the SNPs and the concentration remained significant. Radiological progression was associated with 10 of the same SNPs as the concentration and, furthermore, 14 other SNPs within the same area as those for concentration. However, none of them remained significant after correction for multiple testing. The levels of sclerostin were not related to any of the three available SNPs from the Immunochip data (rs3785806, rs2090019, rs1513670).

## Discussion

In this study, we have shown that there is a clear relationship between RANKL and the Larsen score at baseline and at 24 months and radiological progression at 24 months before and after adjustments for markers of disease activity. Consequently, RANKL concentration can give prognostic information of joint destruction already at baseline predicting the outcome after the first 2 years of disease progression. In a previous study, the ratio of RANKL/osteoprotegerin was shown to be a predictive marker of radiological progression over 11 years as opposed to RANKL alone (22). We can, therefore, in contrast to that study performed on several biomarkers, identify RANKL as a valuable predictor for radiographically detected progression (23). In that study, only free RANKL was detected, while in our study both free and bound RANKL was measured. However, that study was performed over a longer time course, 5 and 10 years, and identified C-telopeptide-1 as a marker (23). RANKL has been concluded as a good biomarker for structural damage of average strength of evidence (24), although further studies on larger cohorts have been suggested.

The concentration of RANKL was particularly increased in anti-CCP2- or RF-positive patients. The concentration of RANKL remained significantly increased in anti-CCP2-positive patients who were RF negative, a finding in line with results reported by others (25). A particularly strong radiological progression was found in patients with the combination of RANKL and anti-CCP2 antibodies. Despite the two factors being significantly related, we were unable to show any interaction between these two factors. The association between ACPA, with or without RF, and radiological progression has been shown in several studies (12, 26, 27). In a previous study on individuals before the onset of symptoms, we found an association between presence of anti-CCP2 antibodies and radiological findings, as measured by the Larsen score at the time of diagnosis of RA years later (27). Also, an increased magnitude of ACPA isotypes has been associated with more radiological damage (28). Both ACPA and RANKL have been shown to affect osteoclasts by promoting osteoclast differentiation (29). Their potential mechanisms of interactions have not been demonstrated although we found a statistical association between the two factors without being able to show a statistical interaction of the two factors. RANKL concentration has been shown to be up-regulated by pro-inflammatory cytokines and is suggested to be of importance for inflammatory bone loss in patients with RA (5). Our findings of relationships between the levels of RANKL and ESR, both on a group and individual level, support a relationship with inflammation. However, there were no significant relationships between CRP or DAS28 and RANKL levels on different time points or as accumulated values (AUC). We were unable to evaluate the potential effects of treatment since almost all patients were on DMARD therapy. Corticosteroid treatment included in the models did not affect the results.

Despite the fact that there was an increased concentration of sclerostin in female patients compared with controls, we did not find a significant relationship between the concentrations or positivity for sclerostin and radiological findings. This was in line with two other recently published studies on radiological findings in RA (30, 31). However, the number of sclerostin-positive patients in this study was low, 18 (4.4%), reducing the strength of the calculated results. Sclerostin levels are related to bone mineral density, which could possibly explain our findings (32). We do not know the bone mineral density of our patients included in this study, but as most of them are postmenopausal with RA, we can assume they will have decreased bone density compared to the controls, e.g., women from the general population.

We have also investigated the gene polymorphisms of the sclerostin (*SOST*) and RANKL genes (*TNFSF11*) in relation to concentrations of sclerostin, RANKL, and to radiological progression. Polymorphisms of 50 of the SNPs for the RANKL gene showed increased concentrations of RANKL and for some of them with radiological findings, although no SNP remained significantly related to the concentration or radiological findings after correcting for multiple testing. In a small study from Japan, one SNP (rs2277438) in RANKL gene was associated with radiographic progression at 2 years (33). We were unable to evaluate this SNP in our study as it was not included in the Immunochip. Risk alleles of genetic variants of *DKK-1* in patients with RA have previously been associated with a more progressive course of joint destruction over time and higher concentrations in serum of functional Dickkopf-1 (34). We could not find a similar relationship for sclerostin even though it is involved in the same pathway as Dickkopf-1. Three SNPs for sclerostin (rs3785806, rs2090019, rs1513670) were not associated to either concentration of sclerostin in plasma or to radiological progression. However, previous studies have shown that several variants in *DKK-1* and other *SOST* were related to structural damage and progression, but we were not able to confirm that findings in our study (35).

In summary, we have shown that measurement of RANKL concentration, on its own, can be a valuable predictor for Larsen score at 24 months and long-term radiological progression irrespective of disease activity. The combination of RANKL and presence of anti-CCP2 antibodies are markers for a more destructive process. The presence of these markers suggests initiation of a more aggressive treatment regimen. Furthermore, we were unable to show that the polymorphisms of the RANKL SNPs were associated with the concentration of RANKL or with the radiological progression. Sclerostin was not related to the radiological findings during the first 24 months.

## Electronic supplementary material

ESM 1 (DOCX 76 kb)
