# Supplementary material for: Receptor activator of nuclear factor kappa-B ligand (RANKL) but not sclerostin or gene polymorphisms is related to joint destruction in early rheumatoid arthritis
Source: Clin Rheumatol. 2017 Feb 11;36(5):1005–12. doi: 10.1007/s10067-017-3570-4 (PMC5400786; doi:10.1007/s10067-017-3570-4)
Supplement: Supplementary file 1 — (DOCX 76 kb) [file 10067_2017_3570_MOESM1_ESM.docx]

Supplementary Table 1. Descriptive data for 407 patients with early rheumatoid arthritis at inclusion and during follow up until 24 months.

| **DAS 28 baseline (mean±SEM)** | 4.7±0.07 |
| --- | --- |
| **DAS 28 6 months (mean±SEM)** | 3.5±0.07 |
| **DAS 28 12 months (mean±SEM)** | 3.2±0.07 |
| **DAS 28 18 months (mean±SEM)** | 3.1±0.07 |
| **DAS 28 24 months (mean±SEM)** | 3.3±0.18 |
| **Swollen Joints baseline median (IQR)** | 6 (7) |
| **Swollen Joints 6 months median (IQR)** | 2 (4) |
| **Swollen Joints 12 m median (IQR)** | 2 (4) |
| **Swollen Joints 18 m median (IQR)** | 1 (4) |
| **Swollen Joints 24 m median (IQR)** | 1 (4) |
| **Tender Joints baseline median (IQR)** | 5 (8) |
| **Tender Joints 6 m (median)** | 2 (4) |
| **Tender Joints 12 m (median)** | 1 (4) |
| **Tender Joints 18 m (median)** | 1 (3) |
| **Tender Joints 24 m (mean)** | 1 (4) |
| **Larsen Score Inclusion (median, IQR)** | 5 (8) |
| **Larsen score 24 months (median, IQR)** | 9 (11) |
